# Evolving a photosynthetic organelle

**DOI:** 10.1186/1741-7007-10-35

**Published:** 2012-04-24

**Authors:** Takuro Nakayama, John M Archibald

**Affiliations:** 1 Department of Biochemistry and Molecular Biology, Canadian Institute for Advanced Research, Program in Integrated Microbial Biodiversity, Dalhousie University, Sir Charles Tupper Medical Building, 5850 College Street, PO BOX 15000, Halifax, Nova Scotia, B3H 4R2, Canada

## Abstract

The evolution of plastids from cyanobacteria is believed to represent a singularity in the history of life. The enigmatic amoeba *Paulinella *and its 'recently' acquired photosynthetic inclusions provide a fascinating system through which to gain fresh insight into how endosymbionts become organelles.

The plastids, or chloroplasts, of algae and plants evolved from cyanobacteria by endosymbiosis. This landmark event conferred on eukaryotes the benefits of photosynthesis - the conversion of solar energy into chemical energy - and in so doing had a huge impact on the course of evolution and the climate of Earth [1]. From the present state of plastids, however, it is difficult to trace the evolutionary steps involved in this momentous development, because all modern-day plastids have fully integrated into their hosts. *Paulinella chromatophora *is a unicellular eukaryote that bears photosynthetic entities called chromatophores that are derived from cyanobacteria and has thus received much attention as a possible example of an organism in the early stages of organellogenesis. Recent studies have unlocked the genomic secrets of its chromatophore [2,3] and provided concrete evidence that the *Paulinella *chromatophore is a *bona fide *photosynthetic organelle [4]. The question is how *Paulinella *can help us to understand the process by which an endosymbiont is converted into an organelle.

## Plastids evolved once, a long time ago

Photosynthetic eukaryotes are a tremendously diverse collection of organisms, from bacterium-sized unicells and giant kelp in the oceans to the plants and trees that inhabit dry land. Multiple rounds of eukaryote-eukaryote endosymbioses have resulted in a tangled web of plastid-bearing lineages [5]. Yet despite this complexity, all plastids appear to trace back to a single ancient endosymbiotic event between cyanobacteria and a heterotrophic host eukaryote. This so-called primary endosymbiosis probably occurred over one billion years ago [6]. The primary plastids of land plants, green algae, red algae and glaucophytes differ tremendously from their presumed cyanobacterial progenitors. What we know for certain is that the majority of the genes present in the endosymbiont were lost or transferred to the host nuclear genome and the protein products of many of these genes are now reimported into plastids by a sophisticated import apparatus (the TIC-TOC complex; translocon complex of the inner and outer chloroplast membranes [1]). Unfortunately, much of the molecular and cellular evolution that accompanied the transition from cyanobacterium to photosynthetic organelle is unclear. A variety of intra- and extra-cellular cyanobacterial symbionts are found in present-day eukaryotes [7], but the details of their host-symbiont relationships rarely tell us anything meaningful about the evolution of plastids. *Paulinella chromatophora *appears to be a remarkable exception.

## *Paulinella *as a window on organellogenesis

**Figure 1:**
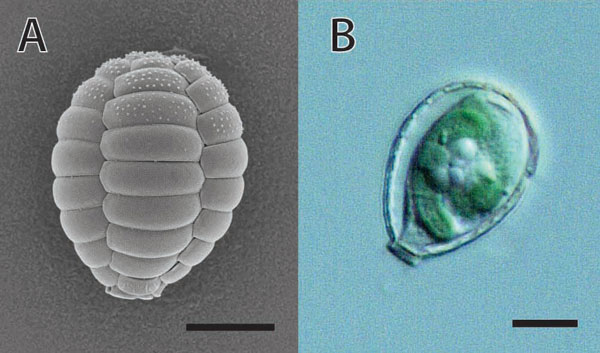
**Scanning electron and light micrographs of photosynthetic *Paulinella *sp**. **(a) **Scanning electron micrograph showing the imbricate scales. **(b) **Light micrograph of a *Paulinella *cell bearing chromatophores. Scale bars = 5 μm.

*Paulinella chromatophora *is an amoeba that belongs to the phylum Cercozoa; it is only distantly related to organisms bearing primary plastids. *Paulinella *cells live in an ovoid, lucid shell made of beautifully arranged silica scales (Figure 1a), and crawl on the bottom of fresh water environments with the help of filose pseudopods. What makes this organism remarkable is the presence of one or two blue-green sausage-shaped chromatophores in its cytoplasm (Figure 1b). Early ultrastructural observations [8] showed that the chromatophore of *Paulinella *shares a suite of characteristics with extant cyanobacteria, particularly members of the genus *Synechococcus*, including the presence of a thick peptidoglycan wall and a similar manner of binary fission. These and other features led to the idea that the *Paulinella *chromatophore is an endosymbiotic cyanobacterium [9], one that is particularly interesting given that it appears incapable of surviving outside of its host [8] and the way it divides and is passed on to daughter cells appears to be orchestrated by the eukaryote in which it resides [10]. The question has long been: is the chromatophore just an endosymbiotic cyanobacterium or is it a photosynthetic organelle [9]? *P. chromatophora *is difficult to grow in the laboratory and progress in addressing this has been slow.

A recent flurry of activity on *Paulinella *was sparked by the work of Marin *et al*. [11]. Working with a highly prized stable culture of *Paulinella chromatophora*, these authors reported the ribosomal DNA (rDNA) sequence of the chromatophore, phylogenetic analysis of which clearly showed that it originated from a member of the cyanobacterial *Synechococcus*/*Prochlorococcus *clade. The chromatophore rDNA sequences show no affinity to those of plastids, indicating that its organelle-like characteristics evolved independently of known plastids and thus could represent another primary endosymbiosis.

To what extent is the chromatophore integrated with the host cell? Key insight has come from the complete chromatophore genome sequences of two *Paulinella *strains [2,3]. Both genomes are approximately 1 megabase pairs (Mbp) in size and contain approximately 850 protein-coding genes. This is significantly reduced compared to the genome of its closest known free-living relative, the cyanobacterium *Synechococcus *WH5701, which is approximately 3 Mbp in size and has 3,346 protein-coding genes. Interestingly, numerous genes essential for cyanobacterial growth have disappeared from the chromatophore genome, including a complete set of genes for the energy-generating tricarboxylic acid cycle, as well as biosynthetic pathways for five amino acids and several cofactors. This explains why the *Paulinella *chromatophore is unable to grow on its own and suggests a significant level of integration with its host, at least at a metabolic level.

## Gene transfer: a necessary but not sufficient step in organellogenesis

The extent and pattern of genome reduction and loss of essential genes in the *Paulinella *chromatophore are very different from that seen in plastids. Plastid genomes are typically <0.2 Mbp in size and most photosynthesis-related genes reside in the nuclear genome of plastid-bearing organisms [12]. In contrast, core genes for photosynthetic activity (which is clearly the main function of the chromatophore) still reside on the chromatophore genome [2,3]. Nonetheless, the degree of genome reduction/gene loss exhibited by chromatophores is roughly comparable to that seen, for example, in the *Buchnera *endosymbionts of aphids, which are not considered organelles (for example, [13]). Therefore, in and of itself, genomic data cannot answer the question of whether the chromatophore is a photosynthetic organelle or not. There has been much debate on the topic [14-16]. It is generally agreed that the existence of a mechanism for the import of host nucleus-encoded proteins is a necessary condition for a subcellular entity of endosymbiotic origin to be considered a true organelle [14,17]. Does such a mechanism exist in *Paulinella*? How much endosymbiotic gene transfer (EGT) has actually taken place?

The answer to the second question has come from analyses of expressed genes in the *Paulinella *nuclear genome. A total of 33 chromatophore-derived nuclear genes have been detected in two *Paulinella *species thus far; in terms of G+C content, these genes are more like nuclear genes than chromatophore genes, and at least some of them have spliceosomal introns (a hallmark of nuclear genes) [2,3,18,19]. Clearly these genes have resided in the host nuclear genome for some time. Interestingly, the products of the majority of these EGT-derived genes are related to photosynthesis, including components of the Photosystem I (PSI) reaction center PsaE, PsaI, and PsaK, strongly suggesting that they function in the chromatophore. But how do they get there? Protein import into canonical plastids is typically mediated by the presence of amino-terminal extensions, referred to as transit peptides, on plastid protein precursors [20], but the deduced amino termini of the *Paulinella *proteins were not obviously longer than their counterparts in cyanobacteria [19].

## Protein import: the last piece of the puzzle

Using biochemical means, Nowack and Grossman [4] have convincingly demonstrated that at least some of the nucleus-encoded, chromatophore-derived proteins in *Paulinella *function in the chromatophore. Specifically, the authors carried out western blot analysis and amino-terminal sequencing to confirm that the product of the nuclear *psaE *gene and two distinct PsaK proteins are assembled into chromatophore-derived PSI. Further, inhibition experiments targeted at cytosolic- and chromatophore-derived ribosomes indicate that the PsaE and PsaK proteins are synthesized by the host's 80S ribosomes. Immunogold electron microscopy using a PsaE-specific antibody revealed a clear accumulation of gold particles on the chromatophore thylakoid membranes, suggesting the existence of a selective protein import system. Even more interesting is the fact that gold particles were also seen decorating the Golgi apparatus of the host, in addition to the chromatophore itself. This result is consistent with the intriguing possibility that the PsaE protein of *Paulinella *is targeted to the chromatophore by an endoplasmic reticulum-Golgi-based system. The host cell secretion system has on multiple occasions been co-opted to function in the targeting of plastid proteins in organisms with secondary plastids (for example, dinoflagellates, euglenophytes and heterokontophytes [1,20]), and also appears to be used to target a minority of proteins to primary plastids [1]. The 'recycling' of pre-existing protein trafficking machinery is thus an emerging theme in the evolution of photosynthetic organelles.

The work of Nowack and Grossman [4] effectively puts to rest doubts over whether the chromatophore of *Paulinella *is an organelle. Yet there is still much to learn. Despite the clear localization of EGT-derived proteins to the *Paulinella *chromatophore, the exact nature of the targeting pathway is still far from clear. Considering currently available bioinformatic, transcriptomic, and amino-terminal protein sequence data, there is no consistent picture as to whether nucleus-encoded, chromatophore-localized proteins such as PsaE and PsaK have amino-terminal extensions that could mediate their transport [4,18], although the presence of signal peptide-like sequences has been proposed [21]. In the case of PsaE at least, transport presumably requires other protein factors and elucidating these factors will be an important next step. Although the PsaE protein of *Paulinella *was shown to pass through the Golgi, the possibility that PsaK and other endosymbiotically derived proteins are targeted to the chromatophore in a Golgi-independent manner cannot be ruled out. Considering the extent of genome reduction and the apparent paucity of transferred genes in the *Paulinella *nuclear genome compared to organisms with canonical plastids, the current state of the chromatophore should be regarded as an early step in organelle evolution. It will thus perhaps not be surprising if its protein import system turns out not to be highly tuned. Ultimately, the extent to which the *Paulinella *chromatophore can shed light on the evolution of canonical plastids will depend on the similarities and differences inferred about their independent evolutionary trajectories.
